# Influence of Different Light Sources on the Biochemical Composition of *Arthrospira* spp. Grown in Model Systems

**DOI:** 10.3390/foods11030399

**Published:** 2022-01-29

**Authors:** Massimo Milia, Francesco Corrias, Piero Addis, Graziella Chini Zitelli, Bernardo Cicchi, Giuseppe Torzillo, Valeria Andreotti, Alberto Angioni

**Affiliations:** 1 Food Toxicology Unit, Department of Life and Environmental Science, University of Cagliari, University Campus of Monserrato, SS 554, 09042 Cagliari, Italy; max_milia@hotmail.it (M.M.); francesco.corrias@unica.it (F.C.); valeria.andreotti@unica.it (V.A.); 2 Biology Section, Department of Life and Environmental Science, University of Cagliari, Via Tommaso Fiorelli 2, 09126 Cagliari, Italy; addisp@unica.it; 3 CNR—Istituto per la Bioeconomia, Via Madonna del Piano 10, Sesto Fiorentino, 50019 Florence, Italy; graziella.chinizitelli@ibe.cnr.it (G.C.Z.); bernardo.cicchi@ibe.cnr.it (B.C.); giuseppe.torzillo@cnr.it (G.T.); 4 CIMAR—Centro de Investigación en Ciencias del Mar y Limnología, Universidad de 17 Costa Rica, San Pedro de Montes de Oca, San Jose 11501, Costa Rica

**Keywords:** *Arthrospira platensis*, *Arthrospira maxima*, spirulina, light sources, food applications, nutraceutics

## Abstract

*Arthrospira platensis* and *Arthrospira maxima* are prokaryotic microalgae commercially marketed as spirulina. The pigments extracted from these algae are widely used for cosmetic and nutraceutical applications. This work aimed to evaluate the influence of three light-emitting lamps (white, orange and blue) on the growth and biomass composition of two strains of *A. platensis* (M2 and M2M) and one of *A. maxima*. The obtained data show strain- and light-dependent responses of the microalgae. In addition, white and orange lights led to a similar overall effect by increasing the levels of chlorophyll *a* and carotenoids. However, exposure to orange light resulted in the highest dry weight (5973.3 mg L^−1^ in M2M), whereas white light stimulated an increase in the carbohydrate fraction (up to 42.36 g 100 g^−1^ in *A. maxima*). Conversely, blue light led to a constant increase in the concentration of phycocyanin (14 g 100 g^−1^ in *A. maxima*) and a higher content of proteins in all strains. These results provide important environmental information for modulating the growth of different spirulina strains, which can be used to address the synthesis of biochemical compounds of strategic importance for the development of new nutraceutical foods.

## 1. Introduction

Spirulina is the trade name of a prokaryotic cyanobacterium belonging to the family Microcoleaceae and the genus *Arthrospira*. It comprises many species, and among these, *Arthrospira platensis* and *Arthrospira maxima* are the most widely used for food, cosmetic and pharmacological applications [1]. These prokaryotes are blue-green algae and grow naturally in the alkaline waters of lakes and confined ponds, preferably in warm areas. *Arthrospira platensis* is found in Africa, Asia and South America. In contrast, wild *Arthrospira maxima* is limited to Central America and has been used for centuries as a food source by various populations [2].

Cyanobacteria are Gram-negative eubacteria and represent the first form of life on earth, responsible for the appearance of the earth’s atmosphere. They are defined as “single-celled organisms that can use photosynthesis” because they can convert solar energy, fixing CO_2_ and N_2_, with a transformation rate ten times higher than that of terrestrial plants [3,4,5]. Thus, they represent an essential ring of the trophic chain of aquatic ecosystems and guarantee the flow of material and energy necessary to maintain heterotrophic organisms. The tiny green filaments of *Arthrospira* are usually coiled in spirals of varying tightness and number, depending on the strain. Proteins represent the main biomass constituent, accounting for almost 60%, whereas carbohydrates account for 15–20% and are represented chiefly by glucan [6]. Lipids account for about 7% of the biomass, and the fatty acid profile is mainly characterized by palmitic (45%), linoleic and γ-linolenic acids. γ-Linolenic acid (GLA) (20%) is the precursor of prostaglandins [6,7,8,9]. Chlorophyll *a* represents 9–12% of the lipid fraction and is chemically pure since cyanobacteria do not contain chlorophyll *b*, as found in microalgae and higher plants. The mineral fraction is characterized by K and P (5.4–8.9%), a high Na content and consistent Ca and Mg levels. In addition, *Arthrospira* contains high levels of vitamin B12 analogues and substitutes, vitamin B complex and fat-soluble vitamins A, K and E [6,10].

Cyanobacteria use a broader spectrum of visible light than green plants (from 540 to 670 nm) to perform photosynthesis from sunlight using photosystems I (PSI, P700) and II (PSII, P680). The process is a light-dependent oxidoreductase reaction involving particular antenna systems that capture the light in each photosystem and convey it to a reaction center, containing two chlorophyll *a* molecules. The intrinsic antenna contains chlorophyll *a* and carotenoids, whereas peripheral antennas contain chlorophylls and phycobilins. Phycobilins, phycocyanin (green-blue color, absorbing red-orange light at a wavelength of 620 nm), phycoerythrin (red color, absorbing at a wavelength of 565 nm), phycoviolobilin (purple color), phycourobilin (yellow color) and allophycocyanin are organized into phycobilisomes. Phycobilisomes exhibit a high capacity for light energy absorption, at wavelengths different from chlorophyll *a*, which is subsequently transferred for photosynthesis [11]. *Arthrospira platensis* phycobilisomes are composed only of C-phycocyanin (C-PC) and allophycocyanin (APC), accounting for 60% of the total protein content [12]. The selective extraction of these pigments allows the manufacture of: natural dyes, fluorescent markers, antioxidants, anti-inflammatory reagents and nutritional ingredients used in cosmetic, health food, nutraceutical and pharmacological applications, with a total market value of phycobiliprotein products of over USD 60 million [13,14,15].

The production of phycobiliproteins (PBPs) and the biochemical composition of the associated biomass can be influenced by the intensity and spectrum of light [16]. Therefore, fluorescent lamps and light-emitting diodes (LEDs) could be utilized as alternative light sources, with different ranges of energy conversion efficiency, to modulate the biomass composition. Preliminary studies conducted at the beginning of the 21st century on the growth of *A. platensis* used colored cellophane paper. In addition, studies with LEDs and fluorescent lamps were recently conducted to investigate the biomass composition and production of PBPs [17,18,19,20].

This work investigated the behavior of three spirulina strains grown in model systems under three different light spectra (white, orange and blue). In addition, growth productivity and the amounts of total lipids, total proteins, total carbohydrates, phycobilins, chlorophyll *a* (Chl*a*) and carotenoids were investigated to evaluate the different performances of the microalgae.

## 2. Materials and Methods

### 2.1. Sample Collection and Processing

Two *Arthrospira platensis* strains, M2 and M2M, and one of *Arthrospira maxima* were obtained from the culture collection of the Institute of BioEconomy (IBE) (National Research Council, CNR Sesto Fiorentino, Italy). Cultures were grown in Zarrouk’s medium in vertical glass columns thermostated at 30 °C and irradiated from both sides with 80 µmol photons m^−^^2^ s^−^^1^ in March 2019 [21]. Culture mixing was conducted by bubbling a mixture of air–CO_2_ (97/3 *v*/*v*) through the culture at 5 L min^−^^1^. The pH was maintained close to 9.5.

As described by Chini Zitelli et al. (2022) [22], an aliquot of spirulina from the bins was diluted to 200/300 mg L^−^^1^ (DW) with the Zarrouk solution and placed in the photobioreactor. Inoculation was conducted under a laminar flow hood, avoiding possible contamination during the transfer. The experimental photobioreactors comprised transparent Pyrex glass tubes equipped with an emery glass stopper with two holes. After three days of acclimatization under the selected spectrum (white, orange or blue), the measurements commenced. The tubes were placed in a parabolic structure that allowed an equal and consistent amount of light exposure (24 h day^−^^1^) of 80 µmol photons m^−^^2^ s^−^^1^ supplied on both sides of the tube, avoiding shadow spots [22].

Three colors of light were applied: (1) white 380–760 nm, maximum 437 nm and 630 nm, (2) orange 615–645 nm, maximum 620 nm, and (3) blue 400–475 nm, maximum 450 nm, at the same light intensity (PFD) of 85–90 μmoL m^−^^2^ s^−^^1^. Sampling (30 mL) was performed daily until the late logarithmic phase, usually reached within seven days of continuous culture. Sterilized distilled water was added daily to compensate for evaporation.

At the end of the experiment, the entire culture was sieved using a 0.25 mm filter, washed with a suitable volume of deionized water, lyophilized and finely ground in a mortar before chemical analysis.

### 2.2. Chemicals

Methanol (MeOH) and chloroform (CHCl_3_) were ultra-residue solvents of analytical grade purchased from Merck (Darmstadt, Germany). Sulfuric acid (95.5%), CaCl_2_, Na_2_CO_3_, CuSO_4_·5H_2_O, Na and K tartrate and PBS pH 7.4 (Na_2_HPO_4_ and NaH_2_PO_4_) were reagent grade (Sigma Aldrich Chemie, Munich, Germany). D-glucose, BSA, tripalmitin and Zarrouk components [21] were of reagent grade and purchased from Sigma Aldrich.

Double-deionized water with a conductivity of less than 18.2 MΩ was obtained with a Milli-Q system (Millipore, Bedford, MA, USA). The Folin–Ciocalteu reagent was purchased from Sigma-Aldrich (St. Louis, MO, USA).

### 2.3. Chemical Physical Analysis

#### 2.3.1. pH

The pH was measured daily with a bench-top pH meter (Hanna Instruments, Rome, Italy) and was maintained around 9; when necessary, it was adjusted by increasing the level of CO_2_.

#### 2.3.2. Dry Biomass Weight (DW)

As described by Chini Zitelli et al. (2022) [22], the mean growth rate (μ) of the crop was calculated with the following formula:

μ (day^−^^1^) = ln (DW_x_ − DW_1_)/T_x_ (1)

DW_x_: dry weight at x day of microalga growth;

DW_1_: dry weight at 1st day of exposure in the photobioreactors;

T_x_: selected time of biomass collection.

### 2.4. Quantification of “Chlorophyll a” and Total Carotenoids

The chlorophyll *a* concentration was measured according to Singh et al. (2016) [23]. Briefly, the gas vacuoles were mechanically broken by pumping the solution with a 5 mL syringe in 2 aliquots before 3 mL of the homogenized solutions was transferred to a 15 mL falcon tube and centrifuged at 3154× *g*, 10 °C for 5 min (Centrifuge 5810 R, Eppendorf AG 22,331 Hamburg). The pellet was suspended in 3 mL of methanol in a vortex for 2 min and left in a thermostatic bath at 70 °C for 3 min; after cooling, it was centrifuged at 3154× *g*, 10 °C for 5 min to precipitate the undissolved cell debris. The supernatant (colored bright green) was analyzed in a UV–Vis spectrometer (Cary 50, Varian Inc.) and calculated according to Chamizo et al. (2020) [24].

Chl *a* mg L^−^^1^ = 12.9447 (A665 − A750) × Volume of methanol (mL)/Volume of sample (mL) (2)

Chl *a*% = (Chl *a* mg L^−^^1^/DW) × 100 (3)

Carotenoid amounts were evaluated with the following formulas adapted from Lichtenthaler [25]:

Carot mg L^−^^1^ = (4.08 × (A470 − A750) − 0.0117 × ChlA mg L^−^^1^) × (Solvent Vol/Sample Vol) (4)

Carot% = (Carot mg L^−^^1^/DW) × 100 (5)

### 2.5. Total Lipids

As described by Chen and Vaidyanathan (2012) [26], 10 mg of lyophilized sample was suspended in 40 μL of PBS 0.05 M (pH 7.4) with 460 μL of NaOH 1 N/MeOH (75/25), and the cells were fragmented in the vortex for 10 min in the presence of glass beads. Subsequently, 1 mL of NaOH 1 N/MeOH (75/25) was added, and the obtained suspension was vortexed for 5 min. Saponification occurred via warming at 100 °C for 30 min.

The solution was cooled down to room temperature and centrifuged at 3154× *g*, 10 °C for 5 min to precipitate cell debris. An aliquot of 1 mL of the solution was transferred to a falcon tube, combined with 3 mL of CHCl_3_/MeOH (2/1) and 0.5 mL of 0.88% KCl, vortexed and centrifuged at 3154× *g*, 10 °C for 10 min to obtain three distinct phases. The calculation of the total lipids was carried out by weight, evaporating 1 mL of the organic solution under a gentle nitrogen stream.

### 2.6. Analysis of Protein Fraction

#### 2.6.1. Total Protein

The extraction and analysis of the proteins was performed according to Lowry et al. [27]. Briefly, 10 mg of lyophilized sample was suspended in 8 mL of deionized H_2_O and cold sonicated for 15 min in the presence of glass beads. After adding 8 mL of NaOH (1 N), the solution was warmed in boiling water for 5 min and cooled under tap water for about 10 min. Total protein analysis was carried out using the Folin–Ciocalteu reagent. Analytical quantification was carried out using a Cary 50 spectrophotometer at 750 nm against a control blank, and a 6-point calibration graph using BSA (bovine albumin) from 20 to 400 mg L^−^^1^. Calibration curves were considered acceptable if r^2^ ≥ 0.997.

#### 2.6.2. Phycocyanin and Allophycocyanin

As described by Siegelman and Kycia (1978) [28], 10 mL of solution was collected from the photobioreactors. Firstly, the gas vacuoles of cyanobacteria were mechanically broken by vigorously pumping the solution with a plastic syringe. Following this, 5 mL was centrifuged at 3154× *g*, 10 °C for 5 min to separate the biomass from the solution. The supernatant was mixed with 5 mL CaCl_2_ (1%, *w v***^−^**^1^) and subjected to manual stirring for 2 min. Then, the solution was subjected to 3 freezing cycles at −80 °C for 1 h and subsequent thawing with warm water. The thermal shock and addition of CaCl_2_ promoted cell lysis and allowed the water-soluble cellular components to pass into the solution. Finally, the tubes were centrifuged at 9000× *g* for 10 min to precipitate unwanted cellular components. The supernatant was analyzed in a UV–Vis spectrometer (Cary 50, Varian Inc.) at 620, 652 and 750 nm.

PC mg L^−1^ = (((ABS 620 − A750) − 0.474 × (ABS 652 − ABS 750))/5.34) × 1000 × (Vol Solv/Vol Sample) (6)

PC% = (PC mg L^−1^/DW) × 100 × Dil (7)

Apc mg L^−1^ = ((ABS 652 − ABS 750) − 0.208 × (ABS 620 − ABS 750)/5.09) × 1000 × (Vol Solv/Vol Sample) (8)

Apc% = (Apc mg L^−1^/DW) × 100 × Dil (9)

### 2.7. Total Carbohydrates

The extraction and analysis of carbohydrates were performed with the phenol-sulfuric acid method, according to Dubois et al. (1956) [29]. Briefly, 2 mg of lyophilized sample was placed in a 15 mL falcon tube with 8 mL of deionized water and sonicated in an ice sonicator bath for 15 min. Following this, 1 mL of the water extract was added to 1 mL of phenol at 5% in water (*w v***^−^**^1^) in a 15 mL falcon tube and vigorously shaken in a vortex for 3 min. After thorough mixing, 5 mL of H_2_SO_4_ (95.5%) was added slowly, with gentle stirring at room temperature for 10 min. The samples were then cooled in freshwater for 20 min and read at a wavelength of 488 nm against a control blank (H_2_O, phenol 5% and H_2_SO_4_).

The 6-point calibration curve of D-glucose (20–120 mg L^−^^1^) was considered acceptable if r^2^ ≥ 0.997.

### 2.8. Statistical Analysis

Analysis of variance (ANOVA) was carried out with the software XLSTAT (Addinsolf L.T.D., Paris, France, Version 19.4); mean comparisons of the effects of treatments were calculated using Fisher’s least significant difference test at *p* ≤ 0.05.

## 3. Results

### 3.1. Chemical Physical Analysis

**Figure 1:**
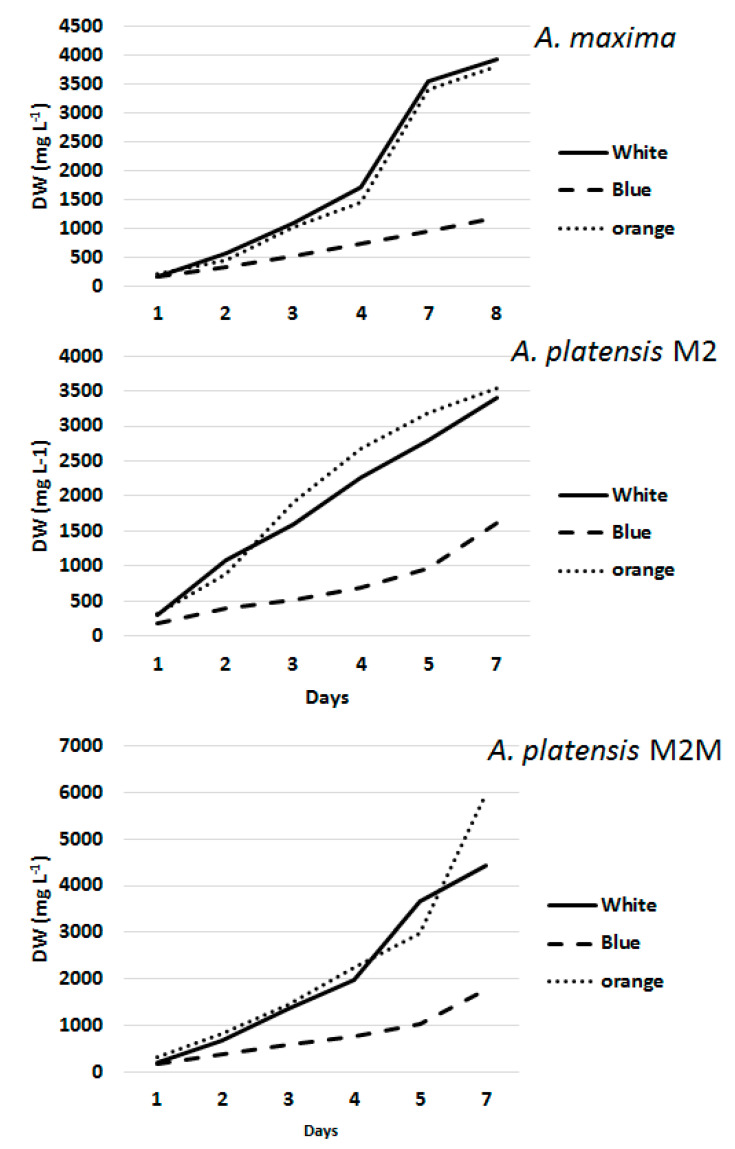
Dry weight (DW) increases after exposing the three selected strains to the irradiation lamps: white, orange and blue.

After one week of culture, the average DW (mg L^−^^1^) reached a maximum with white and orange lights (3919 ± 13 and 4444 ± 29 mg L^−^^1^, mean ± RSD% (relative standard deviation), respectively). In contrast, this result was almost halved (1524 ± 20 mg L^−^^1^) under blue light. *A. platensis* M2M showed the highest values under orange light (5973.3 mg L^−^^1^), followed by *A. maxima* (3813.3 mg L^−^^1^) and *A. platensis* M2 (3546 mg L^−^^1^). *A. maxima* growth was split into three steps of approximately three days each, whereas M2 and M2M showed constant growth (Figure 1).

The growth performance attained under white and orange lights did not differ significantly. The differences in the growth rates appeared to be mainly strain dependent, whereas, when blue light was used, only minor differences were detected among the strains; however, the M2M strain showed a sharp increase after several days under white light.

Analysis of the average growth rate of the strains under different forms of incident light revealed higher growth in the first two days in all experiments, decreasing with different rates on the subsequent days.

### 3.2. Chlorophyll a and Total Carotenoids

**Figure 2:**
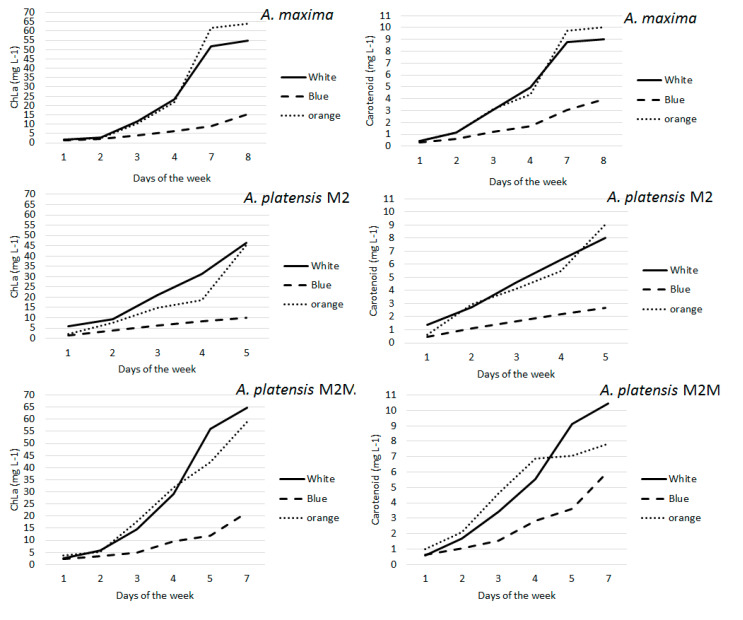
Chlorophyll *a* (Chl *a*) and carotenoid amounts in different *Arthrospira* strains following exposure to white, orange and blue lights.

Chl *a* and total carotenoids exhibited similar behaviors; their levels increased on the second day and reached their maximum after one week. However, carotenoid levels were almost five times lower than those of chlorophyll *a*. *A. platensis* M2M and *A. maxima* showed higher levels of Chl *a* under white and orange lights. Under blue light, the Chl *a* concentration was the lowest in all strains (Figure 2). Among the strains, white light was more effective in M2M, followed by *A. maxima* and M2. Orange light was most effective in *A. maxima*, followed by M2M and M2, whereas blue light was most effective in M2M, followed by *A. maxima* and M2 (Figure 2). Carotenoids were found at the highest levels in M2M, except under orange light, which was more effective in the *A. maxima* strain (Figure 2). Overall, the carotenoid time course demonstrated an increase in total carotenoid levels throughout the whole duration of the experiment, although levels were much lower in cultures grown under blue light, particularly in strain M2.

### 3.3. Total Lipids

Within each strain, different lights led to varying amounts of total lipids. *A. maxima* was found to contain the highest amount of total lipids under white light (9.36 ± 8.25 g 100 g^−1^, mean ± RSD%), followed by orange and blue lights (3.38 ± 13.37 g 100 g^−1^, mean ± RSD%). M2M contained a higher amount of total lipids with orange (8.25 ± 10.51 g 100 g^−1^, mean ± RSD%) and blue lights (7.32 ± 7.17 g 100 g^−1^, mean ± RSD%) and lower values with white light, whereas M2 showed higher values under white light and comparable values under blue and orange light (Table 1).

### 3.4. Analysis of Protein Fraction

#### 3.4.1. Total Protein

All strains showed a lower protein content under orange light, whereas they showed higher values when grown under blue light [30]. The highest value was observed with blue light in M2M, whereas M2 and *A. maxima* showed overlapping values (Table 2).

The amount of protein in the different batches reflected the rate of metabolic activity in the growing cells (Table 2).

#### 3.4.2. Phycocyanin and Allophycocyanin

**Figure 3:**
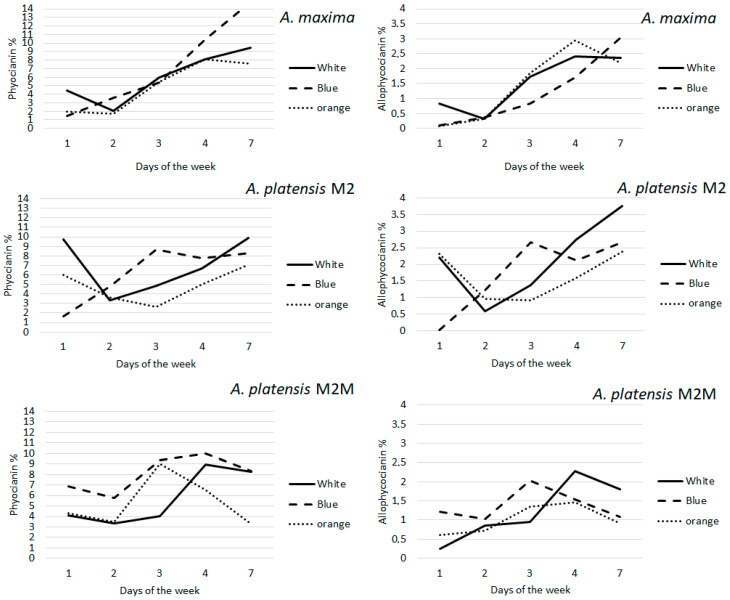
Phycocyanin and allophycocyanin amounts (g 100 g ^−1^ ± RSD DW) after exposing the three selected strains to the irradiation lamps: white, orange and blue.

The phycocyanin content was variable, depending on the light and the strain tested. Blue light strongly influenced the phycocyanin content in *A. maxima*, which was much higher than under white and orange lights. The phycocyanin content in this strain reached 14% of DW under blue light, compared to 9% and 7% under white and orange lights, respectively (Figure 3). Moreover, the phycocyanin content increased more regularly than in M2 and M2M. The allophycocyanin content in this strain showed minor differences between the different light colors tested, ranging between 2.5% and 3% of DW.

In M2, the phycocyanin content ranged from 6% under orange light, up to 10% under blue light, while under white light, it showed an intermediate value (Figure 3). Interestingly, this strain produced the most allophycocyanin, reaching 4% of DW under white light. At the same time, non-significant differences were found between cultures grown under blue and orange lights (Figure 3). The M2M strain started with a high value of phycocyanin relative to the other strains and reached 9% of DW at the end of the experiment under white and blue lights. However, the phycocyanin content was significantly lower under orange light (*p* < 0.001). In this strain, the allophycocyanin content was considerably higher under white light (2% of DW) compared to orange and blue lights (1% of DW).

It is interesting to note that the maximum phycocyanin content (about 10% of DW) with blue light was attained on the third day of culture in strains M2 and M2M. After that, it stabilized to around 9%, while in *A. maxima,* the phycocyanin content rose steadily until 14% without showing any decline.

### 3.5. Total Carbohydrates

The carbohydrate content reached higher levels in *A. maxima* strains under white light (42.36 ± 3.08 g 100 g^−1^, mean ± RSD%), that is, four and two times higher than M2 and M2M, respectively. Under blue light, *A. maxima* strains also reached higher levels of total carbohydrates (21.35 ± 6.98 g 100 g^−1^, mean ± RSD%), that is, about 1.5 higher than in M2 and M2M. In contrast, the M2M strain exhibited the highest total carbohydrate level under orange light (30.17 ± 3.83 g 100 g^−1^, mean ± RSD%), 1.83 and 1.36 higher than M2 and *A. maxima*, respectively (Table 3).

## 4. Discussion

**Figure 4:**
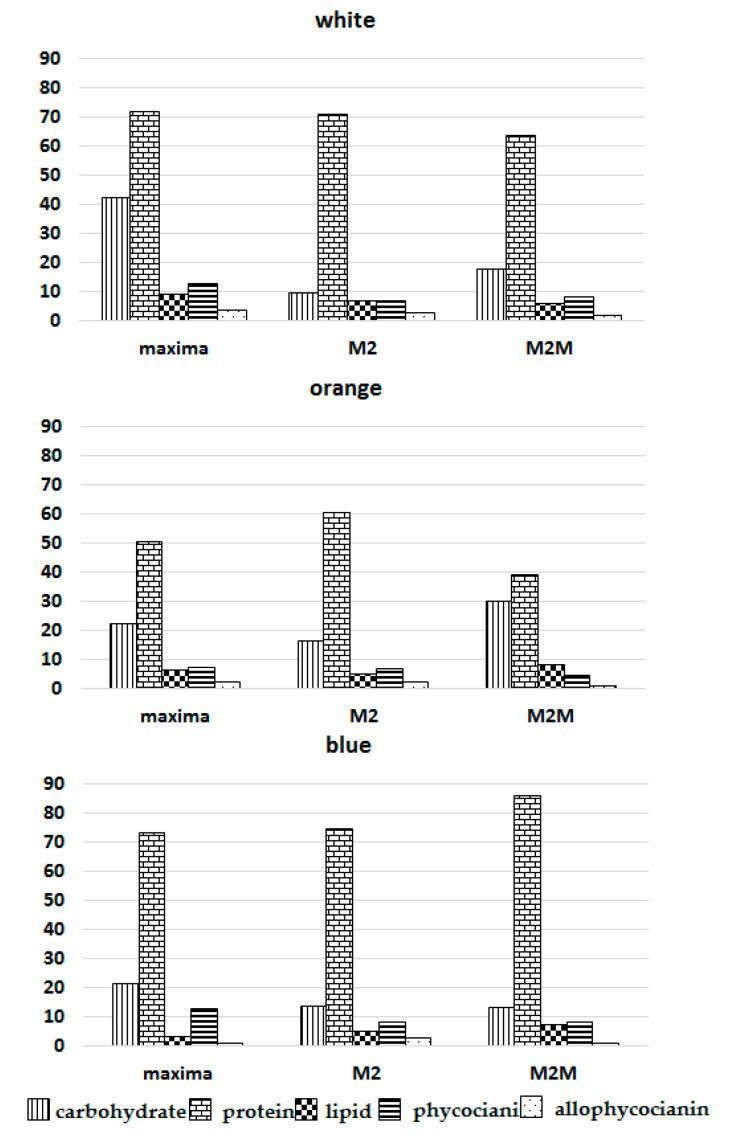
Biochemical composition of the three *Arthrospira* strains following exposure to white, orange and blue lights.

**Figure 5:**
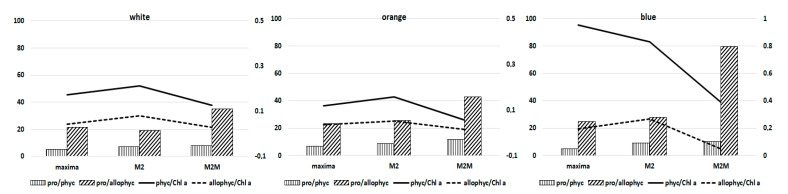
The ratio of protein/phycobilins, and phycobilins/Chl *a*, following exposure to white, orange and blue lights.

*Arthospira* spp. cultures attained the highest dry weight with white and orange lights, while blue light was absorbed less efficiently [16,18,31,32]. The biochemical composition changed during growth, and cultures showed a higher protein content in all strains under blue light, with a similar ratio of protein/phycocyanin in M2 and M2M (Figure 4 and Figure 5).

In contrast, the ratio of protein/allophycocyanin was lower in *A. maxima* and M2M. The mean growth rate of phycocyanin under blue and orange lights was comparable (0.024 h^−1^ blue, 0.026 h^−1^ orange). In contrast, the biomass growth rate strongly diverged (0.014 h^−1^ blue vs. 0.024 h^−1^ orange); according to previous findings, growth was much more limited under blue light than phycocyanin synthesis [22]. In alignment with data in the literature, our experiments showed a lipid fraction ranging from 3 to 9% of DW in all strains. Lipid content is correlated with the temperature, irradiance and availability of nutrients that affect both the lipid content and the fatty acid profile [33,34,35]. High irradiance stimulates an accumulation of triacylglycerols because they may act as energy and electron sinks during stress conditions [33]. It appears that algal cells synthesize triglycerides when the energy input, through carbon assimilation, exceeds the immediate metabolic needs of the cell [36]. In contrast, the presence of a combined nitrogen source increased the cells’ protein fraction, decreasing the levels of lipids and carbohydrates [37].

The data presented here suggest that strains react differently to light colors. The *A. maxima* biomass composition was mainly influenced by white light, whereas that of M2M was more affected by orange light. In contrast, blue light exhibited a lower influence on the accumulation of lipids. The data obtained for carbohydrates agree with previous research findings (15 to 25% DW) [37]. Subjecting *A. maxima* to white light almost doubled the carbohydrate values compared to those observed in other strains after seven days. Most likely, the decrease in nitrogen due to the high dry weight reached by this strain after seven days exhausted the nitrogen supplied with the medium. This behavior is very common in *Arthrospira*, which usually directs excess reducing power toward carbohydrate synthesis during conditions of nitrogen limitation or low-temperature stress [37,38,39]. The amount of total protein varied between 60 and 85% [40,41], except for M2M under orange light (39.09%). This strain attained the highest growth, supported mainly by the synthesis of carbohydrates. The strains exceeding 4000 mg L^−^^1^ in the Zarrouk medium undergo stress related to nitrogen starvation [42]. Indeed, after irradiation with orange light, the M2M strain exceeded this limit by a factor of 1.5 (5973.7 mg L^−1^ vs. 4000 mg L^−^^1^ DW), decreasing the protein content and increasing the carbohydrate content (Figure 4).

According to the literature, blue light positively influences phycocyanin production (Figure 3 and Figure 4) [19,20,43]. In contrast, white light covers a broader range of wavelengths and does not specifically increase phycocyanin production (Figure 3 and Figure 4). Moreover, the ratios of phycocyanin/Chl *a* and allophycocyanin/Chl *a* were higher under blue light than under orange or white light for all strains, indicating that the phycobilin content increased relative to Chl *a* when cells were exposed to blue light (Figure 5).

Blue light stimulates spirulina to produce more phycobilins to compensate for the lack of energy related to the limited range of radiation (400–475 nm), resulting in increased protein production [16,44,45].

The carotenoid profiles showed an increase in carotenoid levels during the whole duration of the experiment, as would be expected according to Chini Zitelli et al. [22].

## 5. Conclusions

Photosynthetic organisms interact with light spectra differently; they have developed several acclimatization strategies to cope with different light sources. *Arthrospira* spp. increase the number of phycobilisomes in limited light conditions, thus significantly altering the protein content. To summarize, in this study, the biomass DW was higher under white and orange lights; moreover, a strong positive correlation between the DW and Chl *a* content was found, ranging from 0.91 to 0.99. The analysis showed that the growth and biochemical composition of the *Arthrospira* strains were strain and light quality dependent. The protein content decreased when the nitrogen source within the medium was exhausted, and higher carbohydrate contents counterbalanced the lower protein level.

Phycobilin production among the different strains was highest in *Arthospira maxima* under blue light. The blue pigment has a powerful antioxidant and anti-inflammatory action and covers an important segment of the health, cosmetic and pharmaceutical markets. Therefore, combining white or orange lights could be used to produce higher amounts of biomass, while blue light could increase phycobilin yields. In conclusion, the exposure of *Arthrospira* cells to different spectra can modulate growth and enhance the production of desired biochemical compounds, particularly proteins, carbohydrates and phycocyanin. The last one seems to be light quality and strain dependent, as in the case of *Arthrospira maxima*. Thus, the light source color is an essential factor that should be considered as a strategic tool when developing new nutraceutical foods.

## Figures and Tables

**Table 1:** Average lipid content (g 100 g^−1^ ± RSD DW) after irradiation of the three strains.

Light	M2	M2M	*A. maxima*
White	7.07 a(a) *	6.11 a(b)	9.36 a(c)
Orange	5.01 b(a)	8.25 b(b)	6.21 b(c)
Blue	5.23 b(a)	7.32 b(b)	3.38 c(c)

* Values in rows (in brackets) followed by different letters differ significantly according to Fisher’s least significant difference (LSD) procedure, *p* ≤ 0.05. Values in columns (without brackets) followed by different letters differ significantly according to Fisher’s least significant difference (LSD) procedure, *p* ≤ 0.05.

**Table 2:** Average protein content (g 100 g^−^^1^ ± RSD DW) in the different experimental conditions of the three strains.

Light	M2	M2M	*A. maxima*
White	71.18 a(a) *	63.71 a(b)	72.14 a(a)
Orange	60.81 b(a)	39.09 b(b)	50.63 b(c)
Blue	74.88 a(a)	85.89 c(b)	73.53 a(a)

* Values in rows (in brackets) followed by different letters differ significantly according to Fisher’s least significant difference (LSD) procedure, *p* ≤ 0.05. Values in columns (without brackets) followed by different letters differ significantly according to Fisher’s least significant difference (LSD) procedure, *p* ≤ 0.05.

**Table 3:** Carbohydrate amounts (g 100 g^−1^ ± RSD DW) under the different experimental conditions of the three strains.

Light	M2	M2M	*A. maxima*
White	9.81 a(a) *	17.75 a(b)	42.36 a(c)
Orange	16.41 b(a)	30.17 b(c)	22.18 b(b)
Blue	13.75 c(a)	13.33 c(a)	21.35 b(b)

* Values in rows (in brackets) followed by different letters differ significantly according to Fisher’s least significant difference (LSD) procedure, *p* ≤ 0.05. Values in columns (without brackets) followed by different letters differ significantly according to Fisher’s least significant difference (LSD) procedure, *p* ≤ 0.05.
